# The Prince of Wales's Hospital Fund Stamps

**Published:** 1897-05-15

**Authors:** 


### THE PRINCE OF WALES'S HOSPITAL FUND STAMPS.

The Jubilee Stamps issued for the benefit of this Fund will be on sale to the public on Tuesday next, May 18th, and can be bought then at all stationers and booksellers, with the exception of the railway bookstalls. They are issued to give small subscribers a handy and convenient form of receipt, and one which they can retain as a memento of the Diamond Jubilee. It is also evident that stamp collectors are largely interested, and spaces for these stamps are being made in many new albums, in addition to the issue of specially-prepared pages for insertion in existing albums, as in that by Messrs. Lincoln. It is impossible to say exactly to whom the initiation of the idea can be credited, but to Mr. Burdett will be due the success of the scheme. The basis of the design selected by the Prince of Wales is taken from no less an authority in art than Sir Joshua Reynolds, whose well-known picture of "Charity," executed for one of the virtues in New College Chapel, Oxford, is the most appropriate design that could have been selected, embodying as it does a beautiful picture, with Mrs. Sheridan as the chief figure.

Valuable assistance has been rendered by Mr. de la Rue and by Mr. Purcell, C.B., the Controller of Stamps at Somerset House, who have taken the greatest interest in the undertaking from its commencement.

After His Royal Highness had approved of the design, the engraving was begun, and proved a very much more serious affair than any one unversed in these details would have anticipated. Such an engraving could only be executed by the most skilled hand. After the matrix had been produced and hardened, each impression had to be separately rolled into the steel plate under a pressure of twenty tons. Each plate contains a double sheet of eighty, and each sheet has to be accounted for as carefully as a bank note, which again entails still greater surveillance.

It is needless to say that the fact of H.R.H. the Duke of York being president of the Philatelic Society insures the greatest interest being taken in these stamps by collectors in all portions of the globe, and that, as a work of art, there will be nothing wanting in them. A large quantity of the issue has already been secured for insertion in the "Queen's Commemoration Bible," and also in the "Queen's Commemoration Prayer and Hymn Book," which are to be published as soon as the stamps are ready, and each of which will contain a stamp. We are informed by the publisher that the greatest interest has been shown all over the country, inquiries having arrived from many places abroad, and telegraphic orders having been received even from South Africa.

His Royal Highness, the President of the Fund, has graciously signified that he will, if possible, personally witness the destruction of the plates from which the stamps are printed; but, in any case, they will be destroyed as soon as the printing of the limited number of the issue is completed, in the presence of the official representatives of the fund, and of Mr. Purcell, C.B., Controller of Stamps. A certificate to this effect will be duly published in accordance with the usual regulations.

Our readers will remember that in the case of the Rowland Hill post-card in 1890 so great was the demand that the value of the post-card advanced no less than 2,500 per cent. We should add that though the trade will be supplied by Messrs. Simpkin, Marshall, Hamilton, Kent, and Co. (Limited), Stationers' Hall Court, London, E.C., the stamps can be bought at all stationers and booksellers, with the exception of railway bookstalls.

